# Histological analysis of age-related degeneration in human female and male knee cartilage and meniscus

**DOI:** 10.1016/j.ocarto.2025.100734

**Published:** 2025-12-18

**Authors:** Amanda Sjögren, Sara Bodahl, Velocity Hughes, Aleksandra Turkiewicz, Anders Aspberg, Iida Hellberg, Ville-Pauli Karjalainen, Neserin Ali, Patrik Önnerfjord, Martin Englund

**Affiliations:** a Lund University, Faculty of Medicine, Department of Clinical Sciences Lund, Orthopaedics, Clinical Epidemiology Unit, Lund, Sweden; b Lund University, Faculty of Medicine, Department of Clinical Sciences Lund, Rheumatology and Molecular Skeletal Biology, Lund, Sweden; c University of Oulu, Faculty of Medicine, Research Unit of Health Sciences and Technology, Oulu, Finland

**Keywords:** Knee, Cartilage, Meniscus, Aging, Degeneration, Histology

## Abstract

**Objectives:**

To assess level of degeneration in human knee cartilage and meniscus in donors without clinically-evident knee arthritis, in relation to age and sex. Further, to investigate the association between cartilage and meniscus degeneration.

**Design:**

Histological sections of knee cartilage and meniscus from 44 deceased donors (ages 18–85 years) without clinically-evident osteoarthritis or inflammatory arthritis were assessed for the level of degeneration using OARSI grades (cartilage) and modified Pauli scores (meniscus). We used Poisson regression models with robust standard errors to investigate the association of age and sex with grades/scores, and the association between cartilage and meniscus degeneration.

**Results:**

Mean [median] (standard deviation) OARSI grade and Pauli score were 2.2 [1.8] (1.2) and 8.2 [8] (2.5), respectively. The mean OARSI grade increased by a factor of 1.19 (95 % confidence interval 1.11 to 1.28) per 10 years of age, and the Pauli score by 1.10 (1.07–1.14). Males had on average 1.29 (0.99–1.69) higher OARSI grades than females, and 1.11 (0.96–1.28) higher Pauli scores. The crude association between Pauli score and OARSI grade was 1.12 (1.05–1.18), while the age and sex adjusted association was 1.06 (0.99–1.13).

**Conclusions:**

Tissue degeneration assessed by OARSI grades and modified Pauli scores increase with age in persons without clinically-evident knee arthritis. However, about half of the elderly donors show only mild degeneration. There was a small association between degeneration and male sex, for both tissues. Cartilage and meniscus degeneration within knees without clinically-evident arthritis were weakly associated.

## 1 Introduction

Osteoarthritis (OA) is a disease with complex etiology, in which the association between structural changes and clinical symptoms remains to be fully understood. It has been reported using MRI and radiography that structural changes in the knee are common in people both with and without risk factors for OA [1]. Furthermore, radiographic evidence of knee OA does not necessarily imply that an individual experiences knee pain [2], a key symptom of OA that often prompts patients to seek medical care [3]. Also, many challenges remain to link changes seen on knee MRI with later OA development [4]. Thus, to better understand knee tissue changes that relate to the disease process, it is important to gain further knowledge on the characteristics of knee tissues in individuals without any clinical history of OA, especially in relation to demographic risk factors such as age and sex [5,6]. There are only a few reports on age- or sex-associated changes in knee components in persons without known clinical knee OA, *e.g.*, articular cartilage (femur, tibia or patella) [[7], [8], [9], [10]], meniscus [[11], [12], [13]], anterior cruciate ligament [14] and chondrocytes [15], where aging suggests to contribute to changes both at the cellular and extracellular level.

Cartilage degeneration in OA has been extensively researched [[16], [17], [18], [19]], and a widely used cartilage histopathological assessment system including early stages of OA progression was developed in 2005 [20] in order to evaluate the severity of the disease. Similarly, the meniscus has been studied for its role in knee OA [21,22], and a dedicated histological scoring and grading system for the meniscus was developed to evaluate the severity of meniscal degeneration [23]. However, it is not well studied how these grades and scores change in tissues across different ages in persons without clinically-evident knee disease. Our hypothesis is that degeneration of cartilage and meniscus increases with age, even in the absence of any clinical history of knee OA.

Thus, the aim of this study was to perform histopathological evaluation of knee articular cartilage and meniscus in deceased human donors across different ages without any clinical history of knee OA or rheumatoid arthritis. Specifically, in a cohort spanning ages 18–85 years, we investigated *i)* the association between age and sex on the OARSI grade of human hyaline cartilage in the medial femoral condyle, *ii)* the association between age and sex on the Pauli score for the human meniscus in the medial posterior horn, and *iii)* the association between cartilage and meniscus histopathology.

## 2 Materials and methods

The ethical review committee of Lund University has approved the sample collection and analysis procedures used in this study (Dnrs: 2015/39; 2016/865; 2019/00323), which were conducted in accordance with relevant guidelines and regulations, including the principles of the Declaration of Helsinki.

### 2.1 Sample cohort

We obtained osteochondral plugs from medial femoral condyles (⌀ = 16 mm) and whole medial menisci from 44 deceased donors (one knee per donor, see Supplementary Figs. S1 and S2 for images of sample retrieval) collected between 2017 and 2022, from the MENIX biobank, Skåne University Hospital, Lund, Sweden. Donors (50 % females, ages 18 to 85 [mean 63, standard deviation 19]) had no clinically-evident knee OA or rheumatoid arthritis, assessed through medical records and information from next of kin. Further exclusion criteria were donors with known drug addiction, hepatitis, or HIV infection, as well as lack of a knee joint. All specimens were obtained within 48 h post-mortem, and were frozen at −80 °C within 2 h of extraction. One female and one male donor (both >65 years old) were wheelchair-users during their final years of life.

### 2.2 Histological preparation

**Fig. 1:**
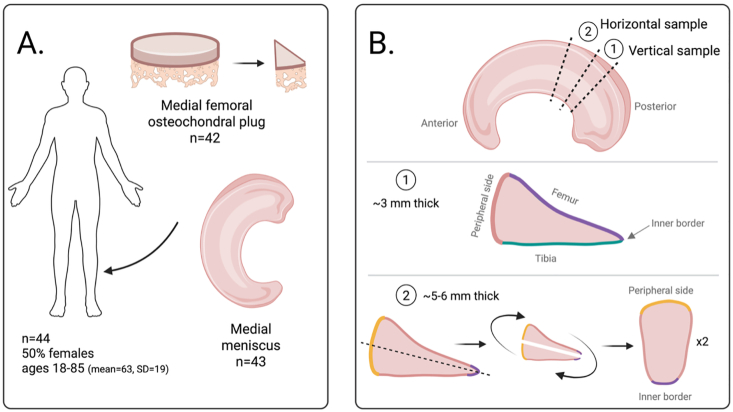
Schematics of sample processing for histopathological analysis. **A.** Medial femoral osteochondral plugs (⌀ = 16 mm) and medial meniscus were retrieved from deceased human donors. A smaller piece from the osteochondral plug was sampled for histology. We excluded one meniscus sample and two cartilage samples due to technical mishaps during sample preparation which likely compromised their tissue quality. **B.** From each meniscus, two types of samples were taken: vertical and horizontal, and we used tissue marking dye to keep track of orientation. The vertical sample was used to assess the femoral (purple) and the tibial (green) surface as well as the inner border. The horizontal sample was cut from the inner border towards the peripheral side (dotted line; video of cutting in Supplementary material 2) after fixation, and was used to assess collagen orientation and cell distribution within the central layer of the meniscus.

We cut smaller samples from each of the osteochondral plugs using a scalpel (Fig. 1A; Supplementary Fig. S1). The samples were directly fixed in 4 % formaldehyde (VWR Chemicals 9713.1000) for 3 days, followed by decalcification with 10 % EDTA for 4 weeks. From each meniscus, one sample for vertical histological sections and one sample for horizontal histological sections was taken from the posterior horn and marked with tissue marking dye (CellPath XMark) to keep track of orientation (Fig. 1B; Supplementary Fig. S2). Both types of meniscus samples were directly fixed in 4 % formaldehyde for 3 days. After fixation, horizontal samples were cut, using a scalpel, to expose the central layer of the meniscus for further sectioning (cutting procedure in Supplementary Video). All cartilage and meniscus samples were dehydrated in solutions of ascending ethanol concentrations, cleared with xylene, and embedded in paraffin. Five μm (cartilage) and four μm (meniscus) tissue sections were cut using a microtome and mounted on SuperFrost Plus slides, and stained with Weigert's iron hematoxylin (Merck CAS 13478-10-9; Sigma-Aldrich H9627), Safranin-O (Sigma-Aldrich S8884) and Fast Green (Sigma-Aldrich F7258). The order in which samples were sectioned and stained was randomized. A detailed histological protocol of the staining procedure can be found in Supplementary Methods. All sections were imaged with an Olympus VS200 slide scanner (20× magnification). We excluded one meniscus sample (male, 78 years old) and two cartilage samples (male, 58 years old; and female, 66 years old) due to technical mishaps during sample preparation which likely compromised their tissue quality.

Supplementary video related to this article can be found at https://doi.org/10.1016/j.ocarto.2025.100734

The following is/are the supplementary data related to this article:

Multimedia component 5 Multimedia component 5

### 2.3 Histopathological assessment

The extent of cartilage degeneration was evaluated using the advanced OARSI histological grading system, developed to assess OA pathology [20]. Grading proceeds inwards from the surface, and if a gradable feature is present at any one site, the sample gets that grade. To assess meniscus degeneration, we utilized the Pauli scoring system [23]. Further details on both assessment systems are available in the Supplementary Methods. In short, OARSI grades range from 0 to 6.5 in half-grade increments, while Pauli scores, which aggregate several features (Supplementary Figs. S3–S6), range from 0 to 18 on a discrete scale. A higher grade and score represent more advanced degeneration of cartilage and meniscal tissue. In this study, however, we did not assess matrix staining in the meniscus samples since all our images had a high intensity of Safranin-O staining, and were not comparable to the representative images in Pauli's work [23]. Therefore, our modified Pauli scoring system generates cumulative scores ranging from 0 to 15. In scoring the vertical sections of menisci (Fig. 1B), we used the same procedure as for cartilage grading. That is, degeneration at any one site was sufficient to assign its corresponding score, *i.e.* the extent of its distribution was not considered. However, for the horizontal meniscal sections (Fig. 1B), we assessed the overall state of collagen orientation and cellularity. Our blinded raters had a high agreement in their scoring of both cartilage and menisci which supports validity of the scoring method and the data.

We excluded portions of the images that were determined to be a result of artifacts introduced during sample preparation (examples in Supplementary Figs. S7 and S8). All images were pseudonymized before grading, and thus the readers were blinded with respect to all donor characteristics and the grading/scoring of the other tissue type. Two raters individually looked through all the histological images before performing consensus grading (cartilage: AS/SB; meniscus: AS/VH, *i.e.*, in total 3 raters were involved). To assess reliability, the same grading procedure was conducted again 8 weeks later for cartilage and 4 weeks later for meniscus, with images pseudonymized again prior to the second round of grading.

### 2.4 Data and statistical analyses

Data analysis was performed in RStudio Version 2024.12.1 + 563. Reliability was analyzed by intra-class correlation coefficients of OARSI grades and modified Pauli scores between the two rounds of consensus grading. ICC estimates and their 95 % confidence intervals (95 % CIs) were calculated based on single measures and agreement of consensus grades/scores [24]. We also assessed the agreement on individual features in the modified Pauli scoring system using Gwet's agreement coefficient, which is recommended for small sample sizes and tie ranks [25]. To analyze how OARSI grades and modified Pauli scores varied with age and sex, we used a Poisson regression model. This model was chosen based on considerations of appearance of the data distribution (with differences appearing to be multiplicative rather than additive). Due to the violation of the assumption that the variance equals the mean, we used robust standard errors [26]. For both cartilage and meniscus, the outcome variable was the mean value of the two consensus readings. Estimates are presented for a 10-year age difference. Further, sex was used as an explanatory variable. Kendall's correlation coefficient was used to estimate correlation between the OARSI grade and the modified Pauli score, and the bias-corrected and accelerated bootstrap 95 % confidence interval was calculated using 9999 replications. Additionally, a Poisson regression model with robust standard errors was used to examine the relationship between the OARSI grade and modified Pauli score, considering both crude and adjusted (age and age + sex) associations. The R-code for the statistical analyses and calculations of intra-class correlation coefficients as well as Kendall's correlation coefficient is available in Supplementary Code.

## 3 Results

Consensus grades for cartilage (OARSI grade) and meniscus (modified Pauli score – individual features and total score) from both rounds of grading are presented in Supplementary Tables 1 and 2, respectively.

### 3.1 Reliability of consensus grading

The ICC estimates for consensus cartilage grading and meniscus scoring were 0.97 (95 % CI 0.94 to 0.98) and 0.84 (95 % CI 0.72 to 0.91), respectively. For further analyses, the mean values of the consensus grades/scores were used for both cartilage and meniscus. In meniscus scoring, the agreement coefficients for individual features were generally good (Supplementary Table 3).

### 3.2 OARSI grades of the cartilage

**Fig. 2:**
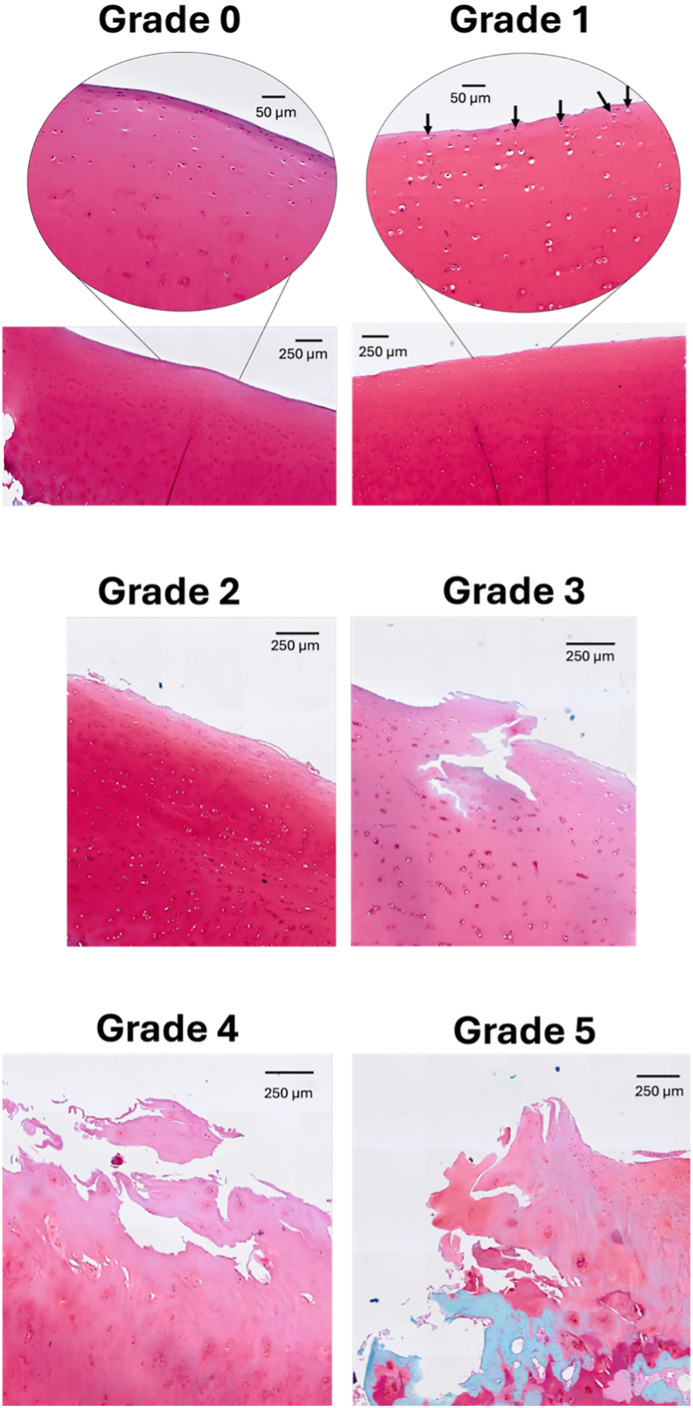
Examples of articular cartilage samples from grades 0 to 5 (no subgrades). Grading of cartilage degeneration in the OARSI system starts from the surface, proceeding inwards. The images presented for each grade received the same grade in both rounds of gradings. **Grade 0:** The surface is smooth, with elongated cells close to the surface. **Grade 1:** The matrix shows swelling and slight superficial fibrillation. Additionally, cells indicate reaction to matrix changes; cells located towards the surface show a slight increase in cell size (arrows). We interpret matrix swelling as absence of cells closest to surface. **Grade 2:** The matrix shows discontinuity in the superficial zone, with loss of cell elongation. **Grade 3:** Fissures extend into the intermediate zone. **Grade 4:** Loss of matrix in the superficial zone. **Grade 5:** Loss of matrix extending to calcified cartilage.

**Fig. 3:**
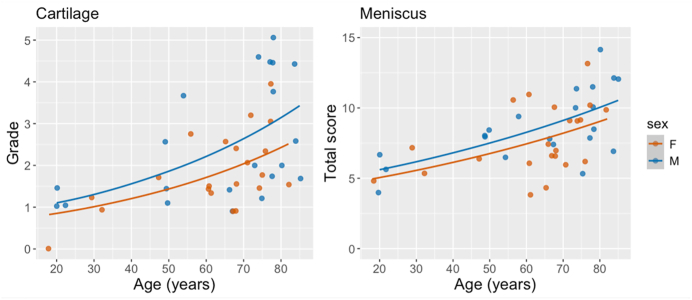
Age (x-axis) and grade/score (y-axis; average of consensus from the two rounds) for cartilage (left) and meniscus (right). Females (F) and males (M) are color coded orange and blue respectively. The lines represent the predicted values from the Poisson models. A jitter function was used to avoid overlapping data points and enhance data visualization.

The OARSI grades in the cohort varied from 0 to 5 (Fig. 2). That is, the three highest grades in the histopathological grading system [20], corresponding to 5.5, 6 and 6.5, were absent. The mean OARSI grade was 2.2 (median = 1.8; standard deviation = 1.2). There was only one donor at each extreme: one with a grade of 0, which was the youngest donor (18 years), and one with a grade of 5, which was among the oldest donors (78 years). All donors below 40 years of age had an OARSI grade less than 2 (Fig. 3). Further, grades between 4 and 5 were only observed in the elderly subset of the cohort. However, the variation in OARSI grades increased with age (Fig. 3). The female and male wheelchair-users (both >65 years old; see *Sample cohort*) both had a grade of 1.5.

### 3.3 Modified Pauli scores of the meniscus

**Fig. 4:**
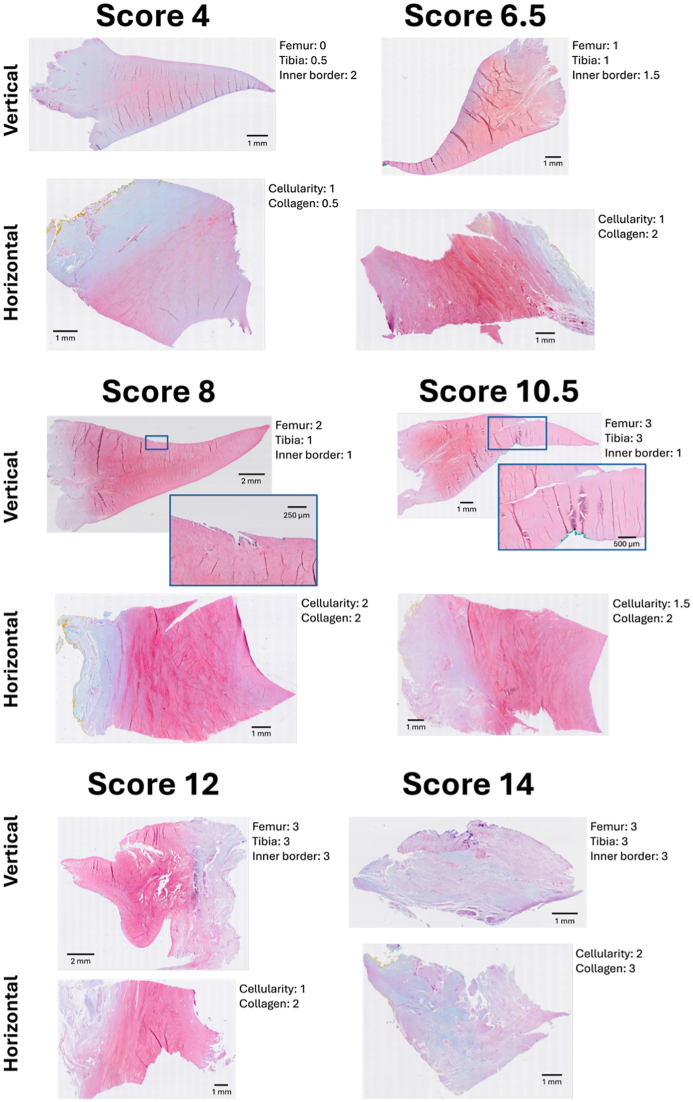
Examples of meniscus samples ranging from modified Pauli overall total score 4 (lowest) to 14 (highest). For each image, the scores for individual features are listed. The scores presented above each pair of images are the average values between the two rounds of scoring.

Using our modified Pauli scoring system (excluding matrix staining), the overall total scores in the cohort ranged from 4 to 14 (Fig. 4). The mean Pauli score was 8.2 (median = 8; SD = 2.5). Sub-scores for both the femoral and tibial surfaces ranged from 0 to 3, cellularity and collagen organization ranged from 0.5 to 2 and 0.5 to 3, respectively, and the inner border ranged from 1 to 3. The same trend of younger donors having the lowest scores, as found in the cartilage samples, was also seen in the meniscus. Similarly, the variation in scores increased with age (Fig. 3). The female and male wheelchair-users (both >65 years old; see *Sample cohort*) had scores of 9 and 8 respectively.

### 3.4 The association between age, sex and degeneration

The OARSI grade increased by a factor of 1.19 per 10 years of age (95 % CI 1.11 to 1.28), *i.e.*, for a 10-year age increase, the mean OARSI grade is expected to be approximately between 10 and 30 % higher. For the meniscus, the modified Pauli score increased by a factor of 1.10 per 10 years of age (95 % CI 1.07 to 1.14). The association between male sex (compared to female) and OARSI grade was 1.29 (95 % CI 0.99 to 1.69), and similar for the modified Pauli score: 1.11 (95 % CI 0.96 to 1.28). This means that males had approximately 30 % higher OARSI grades and about 10 % higher modified Pauli scores, compared to females (Fig. 4).

### 3.5 The association between cartilage and meniscus degeneration

**Fig. 5:**
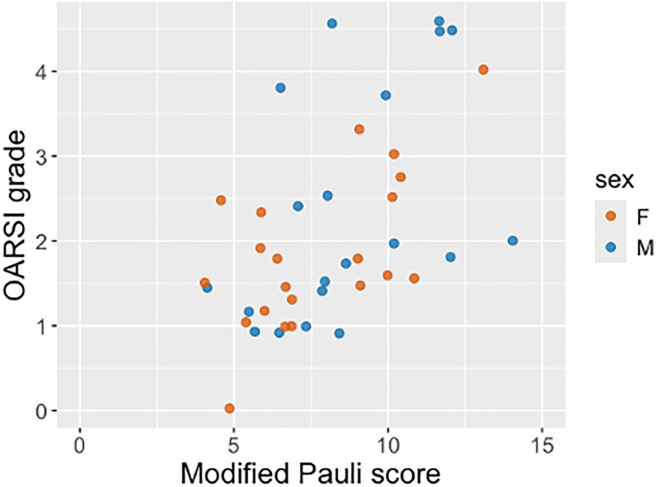
Total modified Pauli score (x-axis; average of consensus from the two rounds) and OARSI grade (y-axis; average of consensus from the two rounds). Females (F) and males (M) are color coded orange and blue respectively. A jitter function was used to avoid overlapping data points and enhance data visualization.

We also analyzed how the OARSI grades and modified Pauli scores varied together (Fig. 5). The Kendall's correlation coefficient between cartilage and meniscus degeneration was 0.40 (95 % CI 0.21 to 0.57), which suggests a positive association, although relatively weak. The crude association between the OARSI grade and modified Pauli score was 1.12 (95 % CI 1.05 to 1.18). The age adjusted association was 1.07 (95 % CI 1.00 to 1.13), while the age and sex adjusted association was 1.06 (95 % CI 0.99 to 1.13). This means that on average, for a one-unit increase in the Pauli score, the OARSI grade is expected to be only ∼6 % higher, and the two tissues are only weakly associated. There was no clear pattern in the relationship between OARSI grades and Pauli scores of individual features (Supplementary Fig. S9).

## 4 Discussion

Our study suggests that, in donors without any history of clinically-evident knee OA or rheumatoid arthritis, increasing age is associated with degeneration of both cartilage and meniscus tissue (Fig. 3). This suggests that aging may lead to histological degeneration similar to clinically-evident OA disease, potentially through mechanisms that stimulate tissue catabolism and cell death [27]. However, our findings suggest that severe cartilage degradation is most certainly not inevitable with aging, but some degree of cartilage and meniscus degradation is to be expected as years go by. Thus, to be able to distinguish when normal aging becomes pathological, that can be labelled as OA, remains challenging, but is also somewhat of a conceptual and philosophical question, including the disease vs. illness perspective.

### 4.1 Cartilage degeneration

From our histological analysis of degeneration in medial femoral knee cartilage, we found that OARSI grades increased with increasing age. Different cartilage features have previously been associated with aging: thinning and fibrillation of patellar cartilage (ages 25–96 years) [28], knee cartilage defect scores (26–61 years) [29] and decrease in thickness (23–74 years) [30], while one study found no association between age and volume for either femoral, tibial (medial and lateral) or patellar cartilage (24–82 years) [9]. However, taken together, these findings indicate that aging may influence several properties of the human knee cartilage. Importantly, none of our most elderly subjects had grades 5.5 or higher and only one had grade 5, which means that aging does not need to be associated with severe cartilage degradation. Cartilage is a tissue that can remain relatively healthy throughout life despite its limited healing and regenerative capacity.

In our study, samples with an OARSI grade >3 exhibited macroscopically observable degeneration, whereas lower grades could only be distinguished histologically. This aligns with previous reports that femoral condyle cartilage from donors without a history of knee arthritis showed slight increase in age-associated surface roughening at the histological level in macroscopically non-eroded cartilage [7]. A previous histological study has indicated that tibial plateau cartilage from OA patients (ages 57–88 years) had an average OARSI grade of 4.1 (SD = 1.3) [31]. In contrast, age-matched femoral condyle samples (age ≥57 years) in our cohort had a lower average OARSI grade of 2.4 (SD = 1.3). However, another study on OA patients found similar average OARSI grades in the femoral medial compartment for slightly younger patients (2.6 [SD = 0.9]; ages 46–78 years) [32]. As previously mentioned, this suggests that aging may be associated with histological degeneration similar to OA in human knee cartilage.

### 4.2 Meniscal degeneration

In our histological analysis of degeneration in the posterior horn of medial menisci, the modified Pauli score had a mean of 8.2 (SD = 2.5). This closely aligns with observations from another study on medial menisci in donors without clinical arthritis, which reported a mean Pauli score of 9.6 (SD = 1.8) [33]. Another study using Pauli scoring for lateral menisci from OA patients (ages 40–94 years) reports a mean Pauli score of 8.0 (SD = 3.8) [34]. Interestingly, age-matched OA-free donors (age ≥40 years) in our cohort had a similar mean Pauli score of 8.6 (SD = 2.4), although we excluded matrix staining as a sub-score. However, another study including both donors without knee arthritis as well as OA patients had median Pauli scores of 11 and 16, respectively, for medial menisci [35]. Similar to hyaline cartilage, aging may be associated with histological degeneration in menisci comparable to OA disease.

In our study, severely degenerated menisci exhibited distorted collagen orientation, and in extreme cases, the histological appearance was more synovium-like with clear loss of collagenous structure. This is supported by a study showing that in menisci without clinically-evident arthritis, dry weight collagen remains stable in normal tissue for individuals over 30 years old, while degenerated areas in age-matched subjects show collagen loss [12]. Further, we could observe an increase in orange color with increasing age by macroscopic examination, similar to what is mentioned in Pauli's work [23], a change likely induced by non-enzymatic glycation [36].

Our results suggest that meniscal structure is already altered at an early age, especially at the inner border, with structural alterations increasing with age. Similarly, a previous MRI study in asymptomatic volunteers (ages 8–62 years) showed that meniscal degeneration increased with age, with early structural changes in many individuals [11]. Further, asymptomatic knees revealed that MR T2 values, which are suggested to be indicative of OA, increased with age in the posterior horn of the menisci, with the highest values observed in the medial compartment [13]. Synovial fluid from joints without knee arthritis and no previous trauma in deceased donors (ages 23–91 years) indicated a decrease in lubricant quality with age [37]. This may be a contributing factor to degeneration of both meniscus and cartilage due to increased frictional force within these tissue compartments.

### 4.3 The association between sex and degeneration

Contrary to our expectations, we found that males had on average more degeneration than females in the studied samples, although with considerable uncertainty (OARSI grades increased by a factor of 1.29 per 10 years of age [95 % CI 0.99 to 1.69]; modified Pauli score: 1.11 [0.96 to 1.28]). We cannot exclude the role of other factors, such as the included persons’ prior physical activity levels, occupational exposures and knee injury. Previous studies suggest that females lose cartilage volume during aging to a higher extent than males [8,38], and cartilage thickness in females over 45 years of age were lower compared to younger females and males of both age groups [39]. Further, a review study found the difference in cartilage volume to be even higher when comparing males with post-menopausal females, with increasing loss of cartilage [40]. However, a study in females without clinically-evident knee OA (40–67 years) investigated patellar cartilage volume change using MRI 2 years from baseline and found surprisingly weak association between age and loss of patellar cartilage volume [10].

### 4.4 The association between cartilage and meniscus degeneration

In subjects without clinically-evident arthritis, we found only a weak association between cartilage and meniscus degeneration, even after adjustments for age and sex. Macroscopic damage to the meniscus has previously been reported to be associated with cartilage loss and joint replacement [41,42]. It might, however, be of importance to differentiate between macroscopic meniscal damage (with potential loss of meniscus function) and microscopic degeneration with otherwise intact structural integrity. One can speculate that long-term degeneration of the meniscal and hyaline cartilage tissues may be relatively independent processes during aging (*e.g.*, different endotypes of degradation), while macroscopic meniscus damage leading to loss of meniscus function contributes to cartilage degeneration to a higher extent due to markedly increased cartilage contact stress. It is also plausible that primary meniscal degradation represents a separate endotype leading to OA as compared to more hyaline cartilage-driven endotypes. However, this warrants further investigation, and it should be emphasized once again that our data were derived from OA-free donors.

### 4.5 Methodological considerations

Two graders conducted consensus grading and scoring, with each grader initially examining each sample individually to perform their first assessments. We believe that subsequent discussion to reach consensus is a reasonable approach for relatively small sample sizes. For larger sample sizes, individual assessments can be more time-efficient, but this approach requires graders to consistently evaluate similar characteristics. Given the inherent subjectivity of grading, we believe that consensus discussions enhanced data quality by allowing for thorough discussion. The reliability of consensus grades/scores for both OARSI and Pauli systems in this study was excellent.

#### 4.5.1 OARSI grading

We observed variation in Safranin-O intensity across grades; however, a more thorough analysis was not conducted, as the OARSI assessment system notes that Safranin-O depletion *may* occur within specific grades, and the primary focus is on the assessment of matrix and cells [20]. One study highlighted the lack of proper assessment of Safranin-O staining as a limitation in the OARSI assessment system, as they observed different patterns of Safranin-O staining even in samples with the superficial zone still present and hence having a low grade [43]. Other studies have emphasized other limitations of the assessment system: the involvement of bone remodeling has been seen in lower grades [31,43], which is not taken into account in the current system. One study reported collagen type II denaturation to start at the cartilage surface and proceed downwards with increasing cartilage Mankin grade [44], which supports the reasoning behind the OARSI assessment system to start from the surface. However, it is essential to acknowledge that alterations deeper within the cartilage are not evaluated with this method.

The suggested threshold for “OA” is defined at an OARSI grade of 1 [20], which, based on our results, may be questioned. According to this definition, we had only one donor without OA in our cohort: an 18-year-old female. Our findings suggest that alterations in the superficial zone cartilage occur as early as 20–30 years of age, which possibly reflects natural aging of cartilage, and not necessarily the OA-related disease processes.

#### 4.5.2 Pauli scoring

The total content of proteoglycans has been reported to increase with age in meniscus and decrease with age in cartilage [36]. In the Pauli scoring system, scoring of proteoglycan content in the meniscus is based on Safranin-O staining intensity. However, all our images exhibited intense Safranin-O staining, warranting the highest score for this feature, rendering it non-comparable to the range presented in the Pauli publication. Thus, we modified the Pauli system [23] by excluding matrix staining as a sub-score. Our modifications have led to lower scores on average and must be considered when comparing the results to other studies. Moreover, the exclusion of Safranin-O staining does shift the focus toward more structural rather than biochemical changes in the meniscus, when compared to the articular cartilage in this study. Upon reviewing the literature on the use of Pauli scores for human meniscus, we observed significant variation in matrix staining across reported images [33,34,45,46], which suggests that the assessment of this feature is very cohort-dependent and might not be well suited for comparisons between independent studies. Enhancing the assessment system could involve presenting a detailed protocol for the matrix staining procedure to facilitate better comparisons between studies.

Cellularity had the lowest agreement among the individual features in Pauli scoring and the scoring of this feature was more challenging than others. Interestingly, differentiating between scores of 0 and 2 was the most difficult (Supplementary Table 3), and other studies have also reported cellularity as having the poorest agreement [47]. Implementing an automated method for scoring cellularity could significantly enhance the reliability of this feature. There ought to be a great potential using AI/machine learning techniques in histological evaluations in general to advance the OA field. Advanced AI techniques have been applied to histological scoring in liver disease and similar approaches could be established for OA [48]. Further, calcium deposits were suggested as an additional assessment in Pauli scoring but were not included in the current study. In a previous study, some degree of calcification was reported in 20 % of 130 meniscal autopsies (age >60 years) from individuals without a clinical history of knee disease [49]. However, menisci from knees without arthritis generally have a low level of calcification compared to OA knees [50], motivating our exclusion of this specific feature.

### 4.6 Limitations

The current study still has several limitations. We assessed histological degeneration of both cartilage and meniscus in human donors without known arthritis. Still, inclusion of donors with true early-stage subclinical OA cannot be excluded (*i.e.* subjects on the pathway to developing symptomatic knee OA). This is, however, the reality in the real world with persons having pre-clinical stages that may, or may not, progress to clinically relevant disease. Important to highlight though, is that we rely on medical records and information from next of kins but lack information about any self-reported history of knee pain or trauma from the deceased donors themselves. Further, the extent of degeneration across the whole cartilage tissue and the whole meniscus is not captured with the histological methodology used. For example, one study mentioned large variations in OARSI grades across the tibial plateau from total knee replacement patients [51]. We sampled cartilage from approximately the same weight-bearing area in the femoral condyle for each donor in our study (Supplementary Fig. S1), but might nonetheless miss degenerated areas at other locations, including the tibia. Another limitation is freezing of the tissue during storing, as the expansion of water in the matrix might impact tissue and cell arrangement at the histological level. While technically possible, using freshly fixed tissue would not have been time-efficient, as gathering a sufficiently large cohort of varying ages would have taken a considerable amount of time. The two wheelchair-users (in their final years of life) had relatively low histological grades of both menisci and cartilage. It is unclear how their limited knee loading may have affected the tissue structure. Finally, manual assessments are prone to subjectivity, as supported by comparisons with other publications that utilize the same assessment systems (see 4.1. Cartilage degeneration and 4.2. Meniscal degeneration).

### 4.7 Conclusion

Our study contributes with new insights into histological cartilage and meniscus degeneration with aging in donors without known clinically-evident knee arthritis. However, based on our results, it is important to highlight that high levels of tissue degeneration are not necessarily inevitable with aging. We found a small association between degeneration in cartilage and meniscus with male sex and a weak association between degeneration in cartilage and meniscus.

## Author contributions

AS, AT, PÖ, and ME were responsible for study conception and design. The experimental work was conducted by AS, SB and VH. Statistical analyses were performed by AS and AT. All authors were involved in the interpretation of the results. Data visualization and manuscript drafting were performed by AS. All other co-authors critically revised the manuscript for important intellectual content and gave final approval of the manuscript for submission.

## Declaration of Generative AI and AI-assisted technologies in the writing process

During the preparation of this work the author(s) used Microsoft Copilot, powered by GPT-4, to improve the quality and readability of the text by asking Copilot to present alternative versions to one or a couple of sentences at a time. After using this tool/service, the author(s) reviewed and edited the content as needed and take(s) full responsibility for the content of the publication.

## Role of the funding source

The funders were not involved in the experimental work, data analysis, writing the manuscript, or publication of this study.

## Conflict of interest

ME reports past (within 3 years) consultancy for Grünenthal Sweden AB and Key2Compliance AB. AT is an associate editor for statistics for the journal Osteoarthritis and Cartilage.
