# Supplementary material for: Histological analysis of age-related degeneration in human female and male knee cartilage and meniscus
Source: Osteoarthr Cartil Open. 2025 Dec 18;8(1):100734. doi: 10.1016/j.ocarto.2025.100734 (PMC12796930; doi:10.1016/j.ocarto.2025.100734)
Supplement: Multimedia component 1 [file mmc1.pdf]

```r
####################
# load necessary packages
pacman::p_load(pacman, rio, dplyr, sandwich, msm, ggplot2, irr, confintr)

# TODO: change to folder location for the file "supplementary.tables.xlsx"
setwd("~/Documents/xx/yy/zz")
# or you can manually set the folder location by:
# Session > Set Working Directory > Choose Directory...

# import cartilage data
df.cartilage <- import("supplementary-tables.xlsx", sheet = 2)
# import meniscus data
df.meniscus <- import("supplementary-tables.xlsx", sheet = 3)

####################
# mean value of consensus grades/scores from round 1 and round 2 will be used
# in the statistical models

# cartilage
# create new data frames for each round
a <- df.cartilage %>% filter(round == 1)
b <- df.cartilage %>% filter(round == 2)
# merge them by sample number
df.cartilage2 <- merge(a, b, by = "sample")
# take the mean value of the columns grade.x (round1) and grade.y (round2)
# then, select only columns of interest and rename them (remove .x)
df.cartilage.mean <- df.cartilage2 %>% rowwise() %>%
          mutate(meanGrade = mean(c_across(c('grade.x', 'grade.y')))) %>%
          select(sample, meanGrade, age.x, sex.x) %>%
          rename(age = age.x, sex = sex.x)
# use age per 10 years in statistical model for easier interpretation
df.cartilage.mean <- df.cartilage.mean %>% rowwise() %>%
          mutate(per10years = age/10)
# remove the temporary data frames a and b
rm(a,b)

# Meniscus (same procedure)
a <- df.meniscus %>% filter(round == 1)
b <- df.meniscus %>% filter(round == 2)
df.meniscus2 <- merge(a, b, by = "sample")
df.meniscus.mean <- df.meniscus2 %>% rowwise() %>%
```

```
          mutate(meanOverallScore = mean(c_across(c('overallScore.x', 'overallScore.y')))) %>%
          select(sample, meanOverallScore, age.x, sex.x) %>%
          rename(age = age.x, sex = sex.x)
df.meniscus.mean <- df.meniscus.mean %>% rowwise() %>%
          mutate(per10years = age/10)
rm(a,b)

####################
# Poisson regression
# the model is the same for both cartilage and meniscus:
# grade/score ~ per10years (age) + sex
# grade and age should be numeric and sex should be set as factor

df.cartilage.mean$sex <- as.factor(df.cartilage.mean$sex)
df.meniscus.mean$sex <- as.factor(df.meniscus.mean$sex)

# one assumption of Poisson regression: the variance equals the mean
mean(df.cartilage.mean$meanGrade)
var(df.cartilage.mean$meanGrade)

mean(df.meniscus.mean$meanOverallScore)
var(df.meniscus.mean$meanOverallScore)
# cartilage nor meniscus fullfils the assumption, we must use robust standard
# errors. https://stats.oarc.ucla.edu/r/dae/poisson-regression/ was a great help
# for R code for Poisson regression with robust standard errors. For more
# detailed explanation, please read the webpage

####################
# cartilage statistical analysis: Poisson regression with robust standard errors

# Poisson regression
m1 <- glm(meanGrade ~ per10years + sex,
                  family = "poisson",
                  data = df.cartilage.mean)

# Robust standard errors
# calculate heteroskedasticity-consistent covariance matrix (to adjust standard
# errors)
cov.m1 <- vcovHC(m1, type="HC0")

# calculate new standard errors
std.err.m1 <- sqrt(diag(cov.m1))
```

```r

# create a table for the poisson model with estimates, robust standard errors,
# p-value, lower confidence level, upper confidence level
r.est.m1 <- cbind(Estimate= coef(m1), "Robust SE" = std.err.m1,
                  "Pr(>|z|)" = 2 * pnorm(abs(coef(m1)/std.err.m1),
                                         lower.tail=FALSE),
                  LL = coef(m1) - 1.96 * std.err.m1,
                  UL = coef(m1) + 1.96 * std.err.m1)

# Transform to rate ratios
# exponentiating the coefficients will transform them into rate ratios
rexp.est.m1 <- exp(r.est.m1[, -3])
# standard errors of transformed coefficients
s.m1 <- deltamethod(list(~ exp(x1), ~ exp(x2), ~ exp(x3)),
                    coef(m1), cov.m1)
rexp.est.m1[, "Robust SE"] <- s.m1

# print estimates, robust standard errors and 95% confidence intervals
rexp.est.m1

####################
# meniscus statistical analysis: Poisson regression with robust standard errors
# look at code for cartilage for explanation (m1)

m2 <- glm(meanOverallScore ~ per10years + sex,
          family="poisson",
          data = df.meniscus.mean)

cov.m2 <- vcovHC(m2, type="HC0")

std.err.m2 <- sqrt(diag(cov.m2))

r.est.m2 <- cbind(Estimate= coef(m2), "Robust SE" = std.err.m2,
                     "Pr(>|z|)" = 2 * pnorm(abs(coef(m2)/std.err.m2),
                                            lower.tail=FALSE),
                     LL = coef(m2) - 1.96 * std.err.m2,
                     UL = coef(m2) + 1.96 * std.err.m2)

s.m2 <- deltamethod(list(~ exp(x1), ~ exp(x2), ~ exp(x3)),
                       coef(m2), cov.m2)

rexp.est.m2 <- exp(r.est.m2[, -3])
```

```
rexp.est.m2[, "Robust␣SE"] <- s.m2

# print estimates, robust standard errors and 95% confidence
    intervals
rexp.est.m2

####################
# intraclass correlation coefficients for OARSI grade and
   overall modified
# Pauli score

df.cartilage2 %>% select(grade.x, grade.y) %>%
          icc(model = "twoway",
              unit = "single",
              type = "agreement")

df.meniscus2 %>% select(overallScore.x, overallScore.y) %>%
          icc(model = "twoway",
              unit = "single",
              type = "agreement")

####################
# comparison of grade and score for the two tissues
# Kendall's correlation coefficient

# first, merge cartilage and meniscus data together
df <- merge(df.cartilage.mean, df.meniscus.mean, by = "
   sample") %>%
          select(sample, meanGrade, meanOverallScore, sex.x,
               age.x) %>%
          rename(cartilageGrade = meanGrade,
                 meniscusScore = meanOverallScore,
                 sex = sex.x, age = age.x)

# second, calculate Kendall's correlation coefficient
ci_cor(df$cartilageGrade, df$meniscusScore,
       method = "kendall",
       type = "bootstrap",
       boot_type = "bca",
       seed = 1234)


####################
# Poisson regression with robust standard errors
# look at code for cartilage for explanation (m1)

# Crude model
m3 <- glm(cartilageGrade ~ meniscusScore,
          family="poisson",
```

```
          data = df)

cov.m3 <- vcovHC(m3, type="HC0")

std.err.m3 <- sqrt(diag(cov.m3))

r.est.m3 <- cbind(Estimate= coef(m3), "Robust␣SE" = std.err.m3,
                  "Pr(>|z|)" = 2 * pnorm(abs(coef(m3)/std.err.m3),
                                        lower.tail=FALSE),
                  LL = coef(m3) - 1.96 * std.err.m3,
                  UL = coef(m3) + 1.96 * std.err.m3)

s.m3 <- deltamethod(list(~ exp(x1), ~ exp(x2)),
                    coef(m3), cov.m3)

rexp.est.m3 <- exp(r.est.m3[, -3])

rexp.est.m3[, "Robust␣SE"] <- s.m3

# print estimates, robust standard errors and 95% confidence intervals
rexp.est.m3


# Adjustment for age
m4 <- glm(cartilageGrade ~ meniscusScore + age,
          family="poisson",
          data = df)

cov.m4 <- vcovHC(m4, type="HC0")

std.err.m4 <- sqrt(diag(cov.m4))

r.est.m4 <- cbind(Estimate= coef(m4), "Robust␣SE" = std.err.m4,
                  "Pr(>|z|)" = 2 * pnorm(abs(coef(m4)/std.err.m4),
                                        lower.tail=FALSE),
                  LL = coef(m4) - 1.96 * std.err.m4,
                  UL = coef(m4) + 1.96 * std.err.m4)

s.m4 <- deltamethod(list(~ exp(x1), ~ exp(x2), ~ exp(x3)),
                    coef(m4), cov.m4)

rexp.est.m4 <- exp(r.est.m4[, -3])

rexp.est.m4[, "Robust␣SE"] <- s.m4

```

```
# print estimates, robust standard errors and 95% confidence intervals
rexp.est.m4



# Adjustment for age and sex
m5 <- glm(cartilageGrade ~ meniscusScore + age + sex,
          family="poisson",
          data = df)

cov.m5 <- vcovHC(m5, type="HC0")

std.err.m5 <- sqrt(diag(cov.m5))

r.est.m5 <- cbind(Estimate= coef(m5), "Robust SE" = std.err.m5,
                  "Pr(>|z|)" = 2 * pnorm(abs(coef(m5)/std.err.m5),
                                         lower.tail=FALSE),
                  LL = coef(m5) - 1.96 * std.err.m5,
                  UL = coef(m5) + 1.96 * std.err.m5)

s.m5 <- deltamethod(list(~ exp(x1), ~ exp(x2), ~ exp(x3), ~ exp(x4)),
                    coef(m5), cov.m5)

rexp.est.m5 <- exp(r.est.m5[, -3])

rexp.est.m5[, "Robust SE"] <- s.m5

# print estimates, robust standard errors and 95% confidence intervals
rexp.est.m5
```
