# Supplementary material for: Histological analysis of age-related degeneration in human female and male knee cartilage and meniscus
Source: Osteoarthr Cartil Open. 2025 Dec 18;8(1):100734. doi: 10.1016/j.ocarto.2025.100734 (PMC12796930; doi:10.1016/j.ocarto.2025.100734)
Supplement: Multimedia component 2 [file mmc2.docx]

**Supplementary figures**

**Sample retrieval**

Representative images of sample retrieval of cartilage (Figure S1) and meniscus (Figure S2).


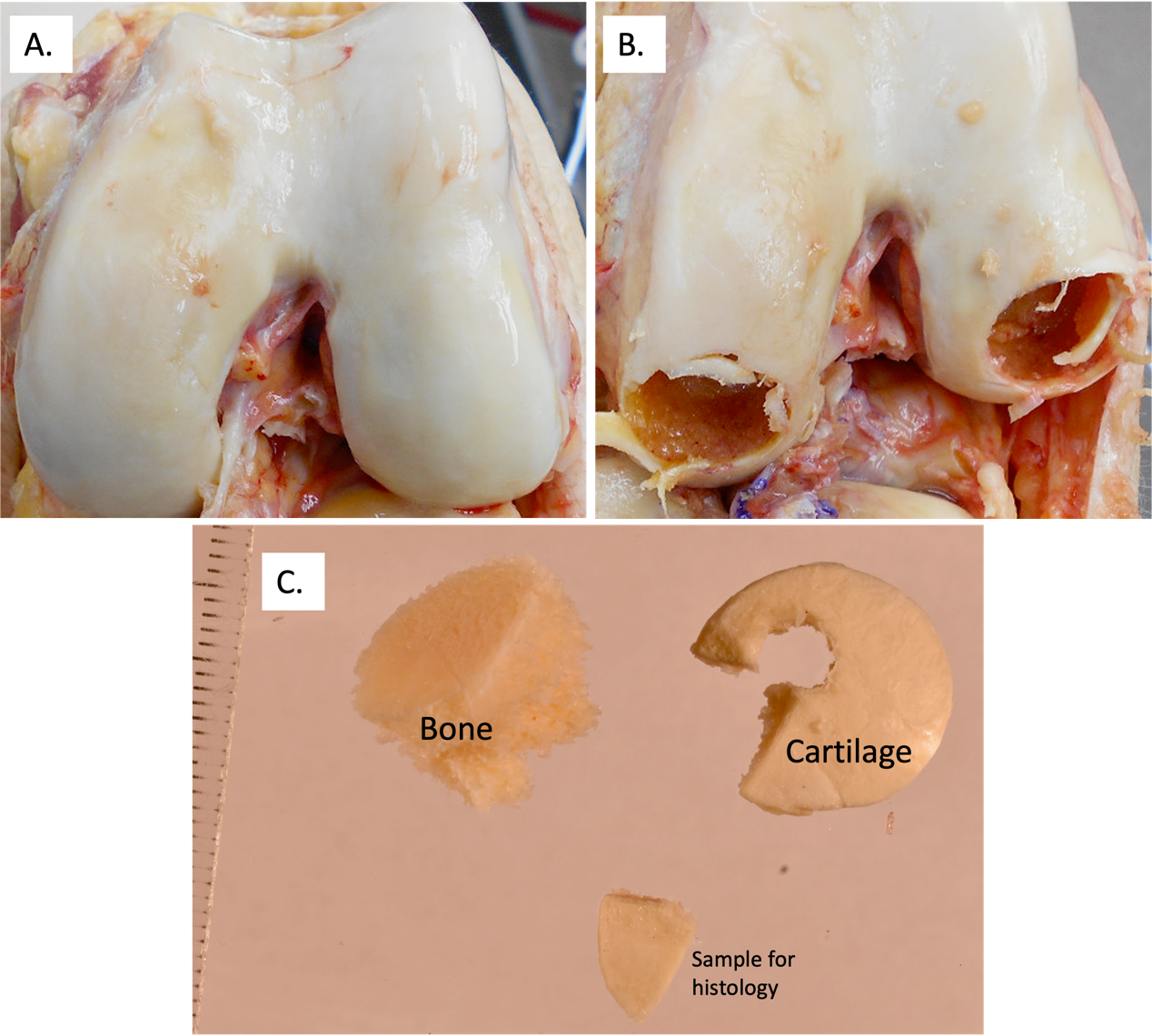


**Figure S1:** Cartilage sample retrieval. **A.** Femoral condyle before the 16 mm plug was taken from the deceased donor. **B.** Femoral condyle after the osteochondral plug was taken. All plugs were taken from the approximate same position on the condyle. **C.** We often remove part of the bone to be able to cut smaller samples using a scalpel or a punch. The cartilage surface is seen in the part called “*Cartilage”*; the hole is from a sample taken for another study. “*Sample for histology”* is a representative sample that was used for cartilage histology in this study. A ruler with 1 mm between each line can be seen to the left.

**
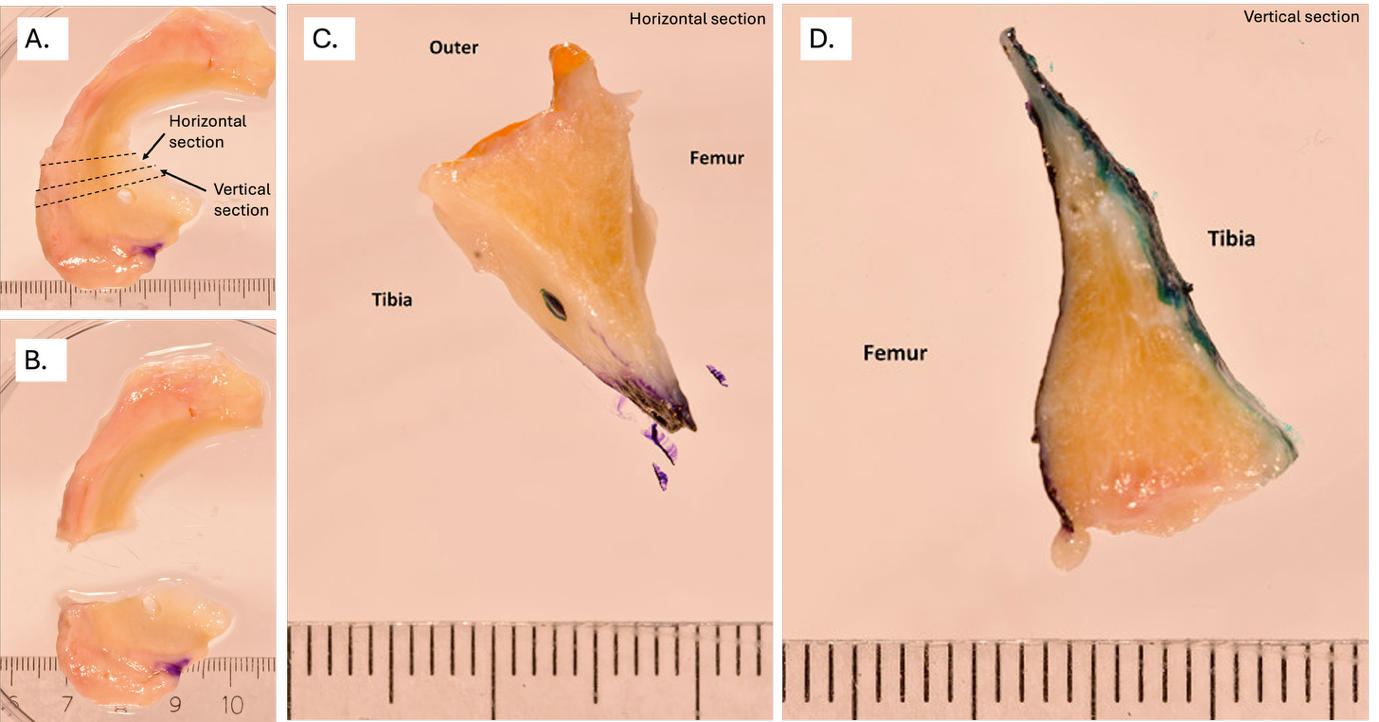
**

**Figure S2:** We receive whole menisci from deceased donors, with the posterior part marked with ink. A ruler with 1 mm between each line can be seen in the bottom of each image. **A.** The whole meniscus before cutting, with the femoral side facing up. The hole is from a sample taken for another study. **B.** The meniscus after sampling for histology. **C.** The extracted sample for horizontal sections with tissue marking dye (see supplementary material 2 for a video of the cutting procedure used to expose the central layer of the meniscus). **D.** The extracted sample for vertical sections, labeled with tissue marking dye.

**Examples of meniscus scores of individual features**

Vertical sections:

- Femoral and tibial surface (Figure S3)
- Inner border (Figure S4)

Horizontal sections:

- Cellularity (Figure S5)
- Collagen organization (Figure S6)

**
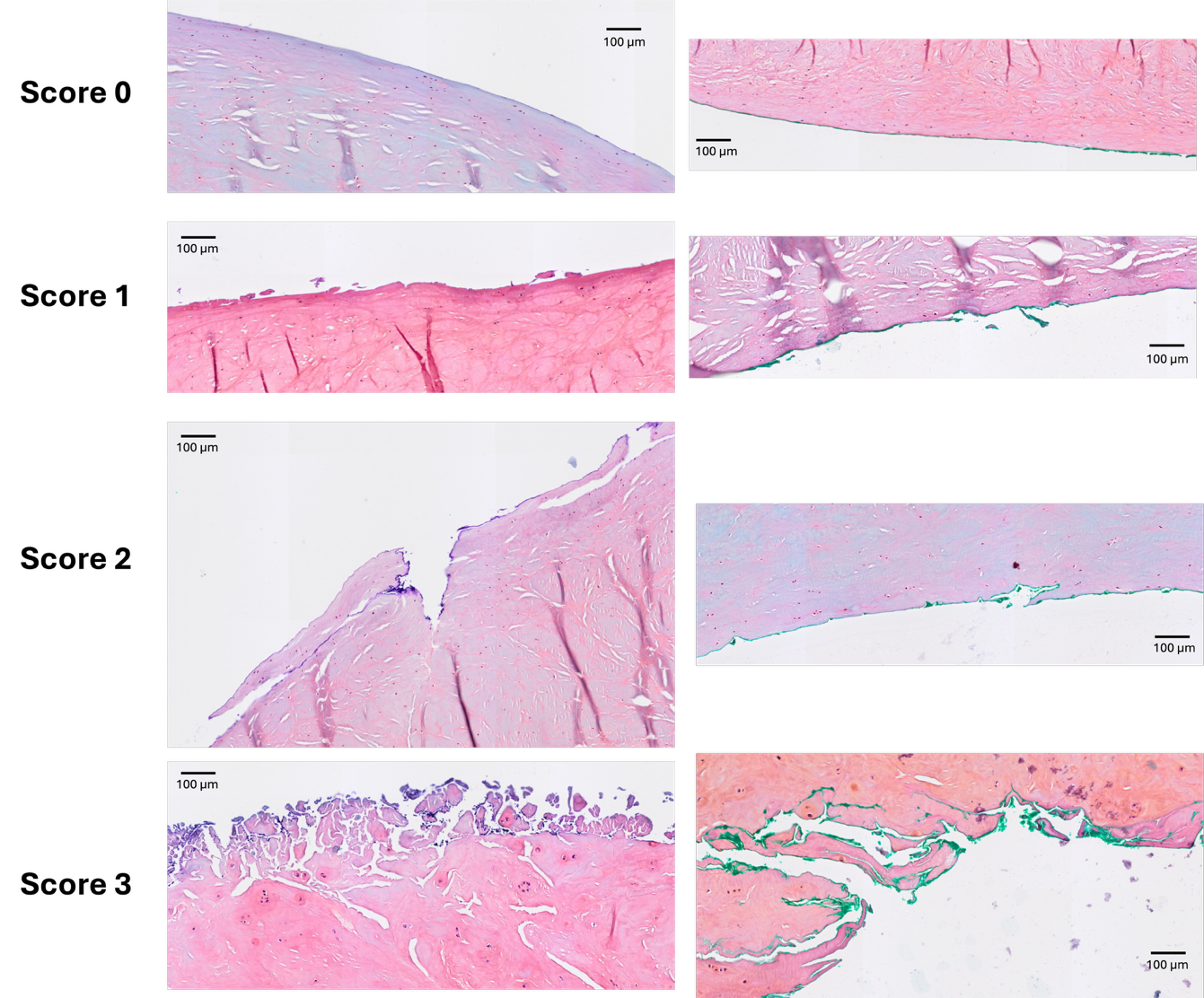
**

**Figure S3:** Examples of femoral (left) and tibial (right) surface scores 0 to 3. The scores were the same in both rounds of scoring. **Score 0:** smooth surface. **Score 1:** Slight fibrillation of surface. **Score 2:** More fibrillation and clefts. **Score 3:** Severe disruption of surfaces.


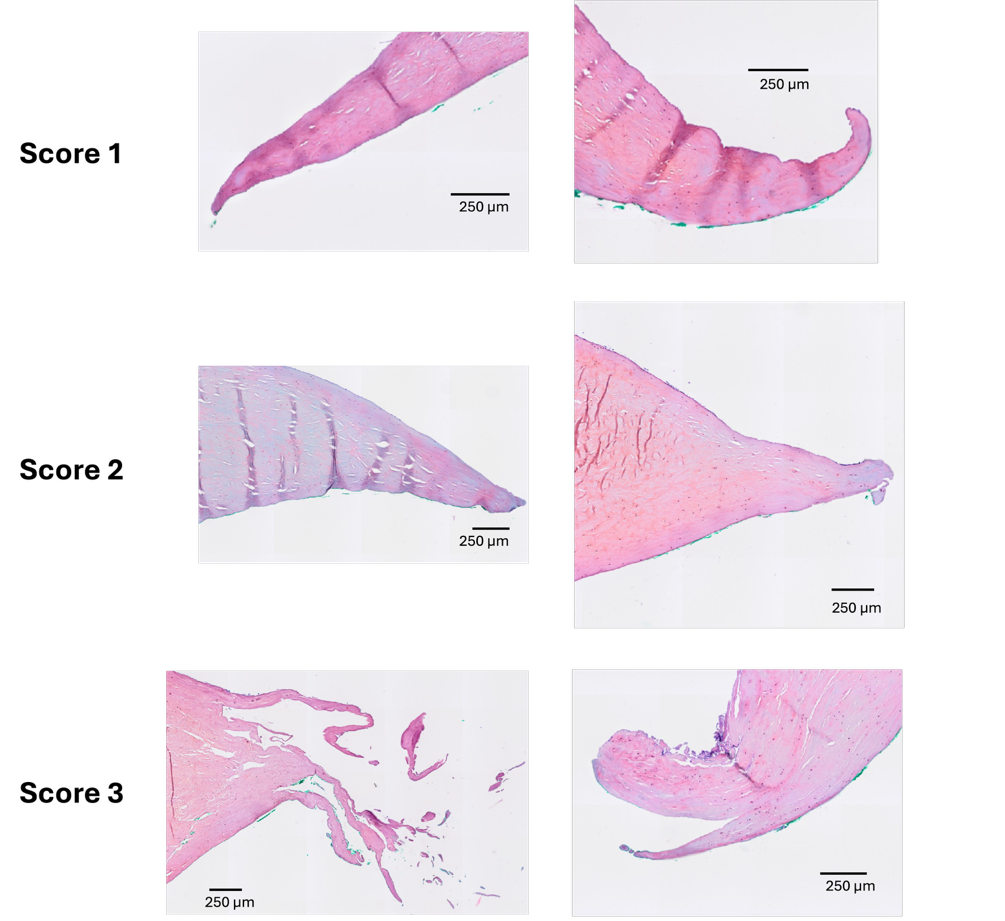


**Figure S4:** Two examples of each score 1 to 3 for inner border. The scores were the same in both rounds of scoring and there was no score of 0 in the cohort.

**
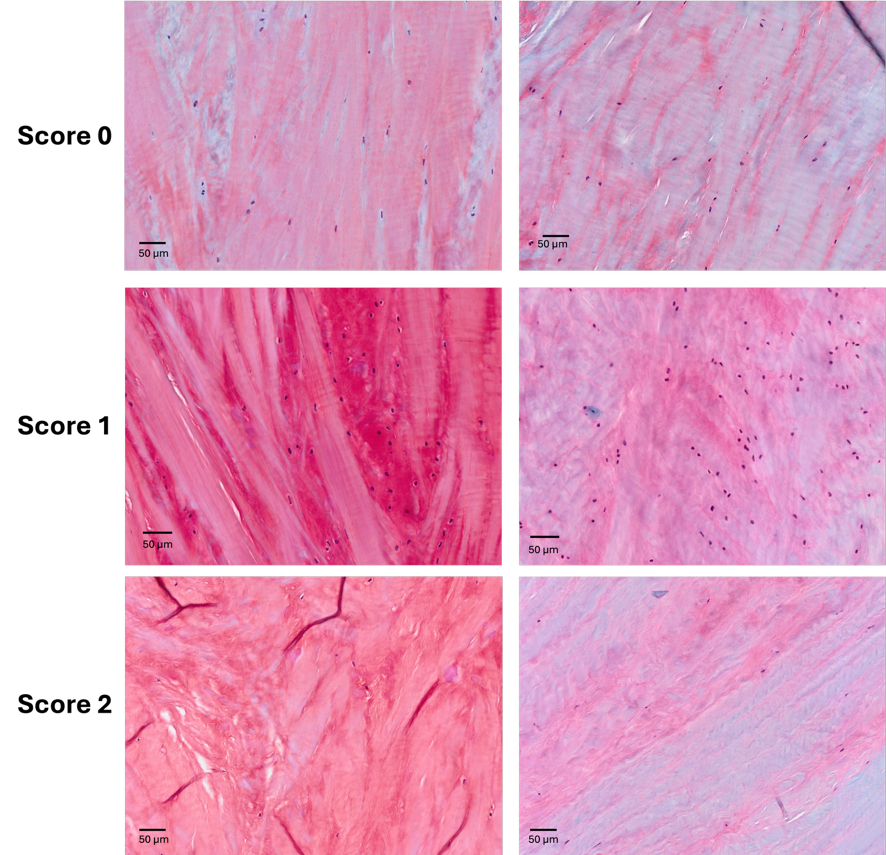
**

**Figure S5:** Two examples of each score 0 to 2 (in either of the two rounds of scoring) for cellularity. For assessment of cells, we focused on the central layer of the meniscal tissue, excluding areas *i)* close to the inner border and *ii)* close to the synovium. There was no score 3 when calculating the average between round 1 and round 2. **Score 0:** normal appearance of cells. **Score 1:** hypercellular regions. **Score 2:** hypocellular regions.

**
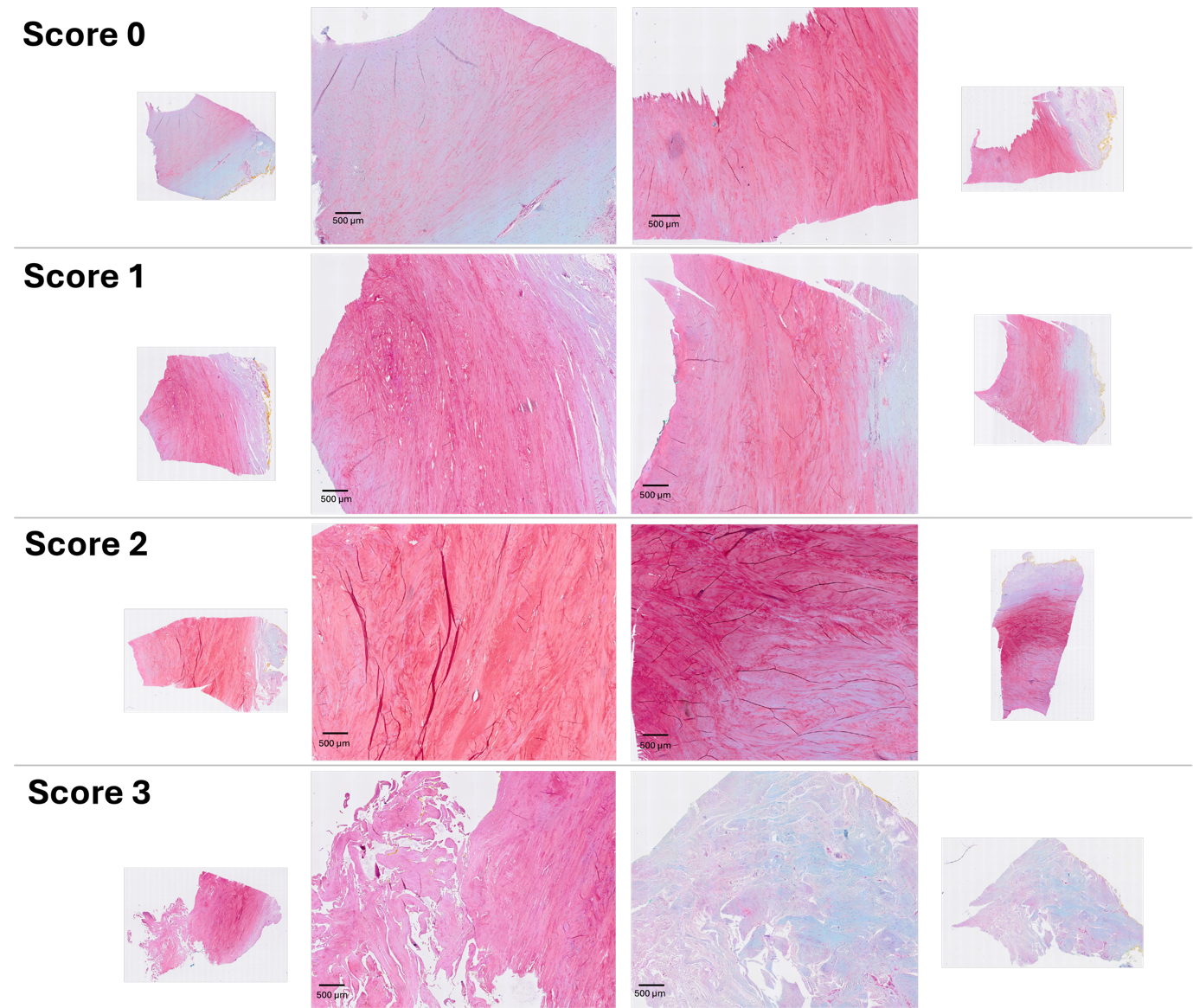
**

**Figure S6:** Two examples of each score 0 to 3 (in either of the two rounds of scoring) for collagen orientation. For assessment of collagen orientation, we focused on the central layer of the meniscal tissue, excluding areas *i)* close to the inner border and *ii)* close to the synovium. The smaller images display the entire section, while the larger images provide a close-up view to enhance the visualization of collagen orientation**. Score 0:** intact matrix. **Score 1:** Some unorganized collagen fibers, but most are organized. **Score 2:** Most collagen fibers are unorganized. **Score 3:** Separation and unorganized fibers. We interpret the sample of score 3 to the right to have very advanced degeneration, with synovium-like tissue present, which can explain the lack of Safranin-O stain.

**Examples of regions excluded due to presumed distortion from sample preparation**

Examples of artefacts that we interpret as introduced during sample preparation (Figures S7 and Figure S8).


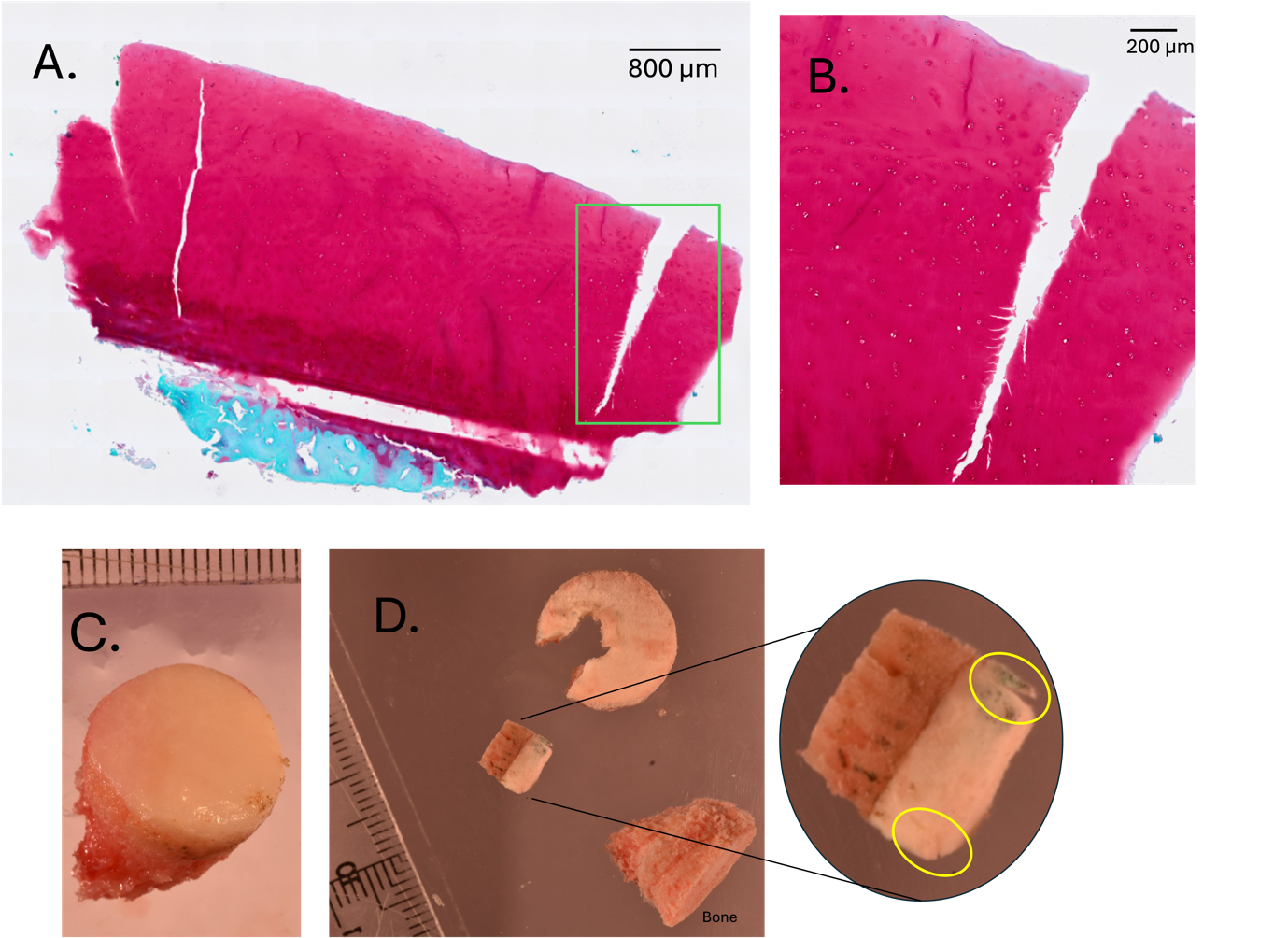


**Figure S7:** Cartilage sample from a 49-year-old male. **A.** Histology image showing two large cracks. **B.** Enlarged view of the crack to the right. The absence of an obvious cell reaction near the crack suggests it is an artifact from sample preparation. **C.** Image of the whole plug (⌀=16 mm) from the same donor, taken after initial retrieval, displaying smooth cartilage. **D.** Tissue documentation image taken while cutting the sample for histology. The zoomed-in view of the histological sample reveals small cracks on both sides, likely resulting from the cutting process.


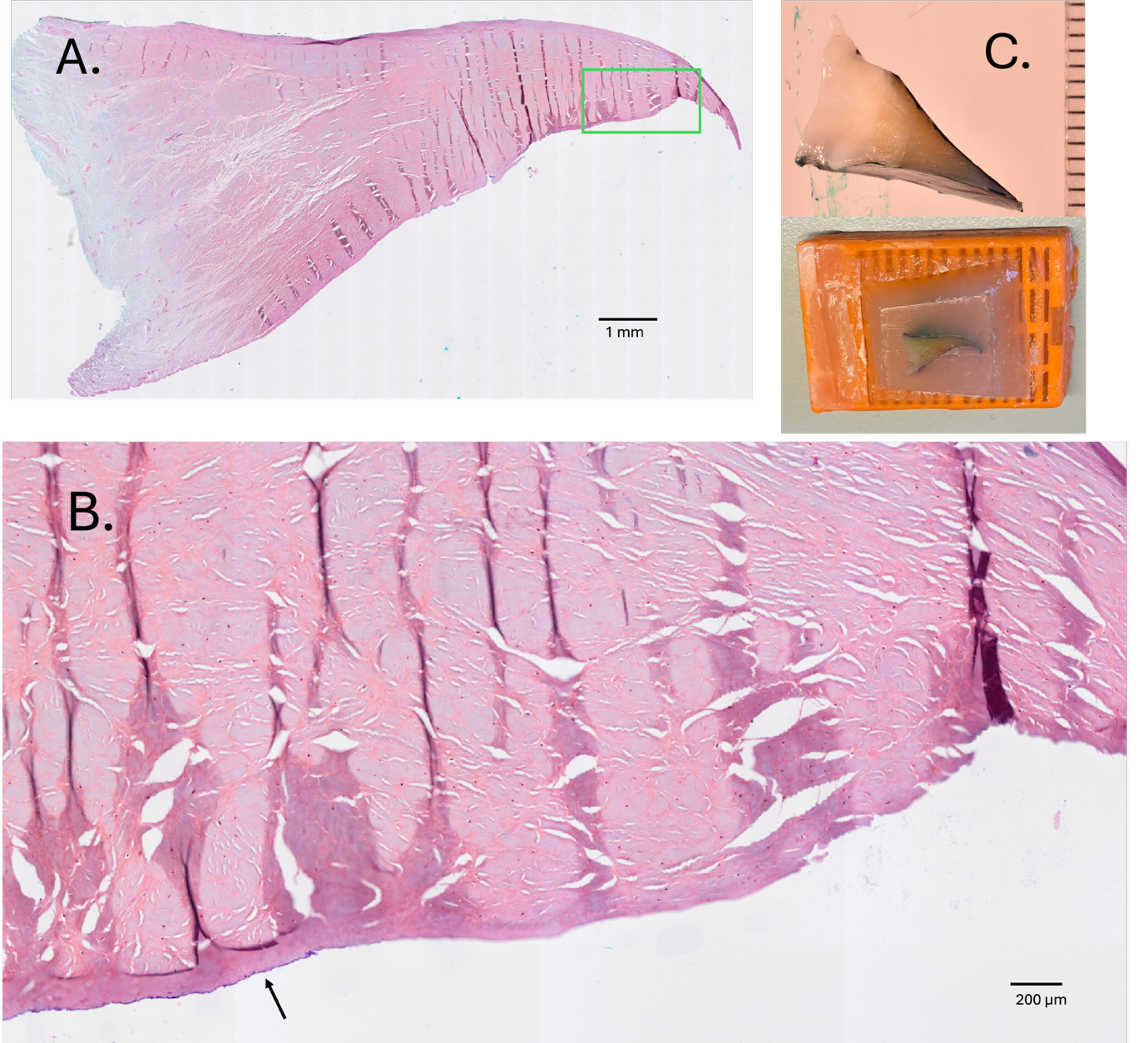


**Figure S8:** Meniscus sample from a 32-year-old female. **A.** Vertical histology image. **B.** Enlarged view of the area within the green rectangle in A, highlighting a region with loss of tissue marking dye. Purple tissue marking dye is visible in part of tissue surface (arrow). **C.** Tissue documentation image from sample preparation (above), and an image of the sample within its paraffin block (below). Both the femoral and tibial surfaces of the meniscus are marked with tissue marking dye, indicating that the surface with missing dye is likely an artefact caused by microtome sectioning (loss of tissue).

**OARSI grades vs Pauli individual feature scores**


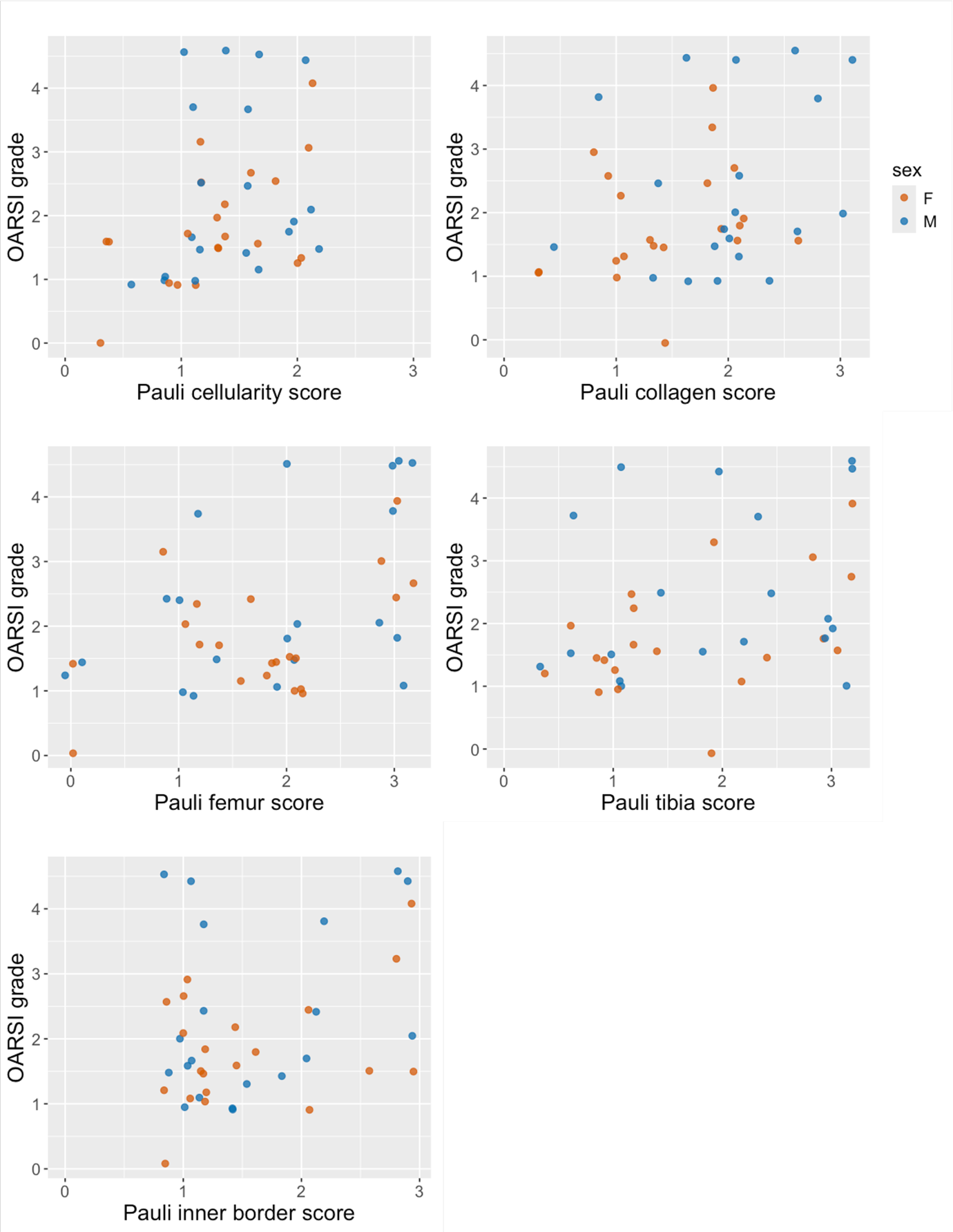


**Figure S9:** Images displaying Pauli scores of individual features (x-axes; mean of consensus from the two rounds) and OARSI grade (y-axes; mean of consensus from the two rounds). Females (F) and males (M) are color coded orange and blue respectively. A jitter function was used to avoid overlapping data points and enhance data visualization.
