# Supplementary material for: Histological analysis of age-related degeneration in human female and male knee cartilage and meniscus
Source: Osteoarthr Cartil Open. 2025 Dec 18;8(1):100734. doi: 10.1016/j.ocarto.2025.100734 (PMC12796930; doi:10.1016/j.ocarto.2025.100734)
Supplement: Multimedia component 3 [file mmc3.docx]

**Supplementary methods**

**Cartilage grading**

In the OARSI assessment system (1), images are first graded from 0 to 6 on a discrete scale. We further used the advanced grading system: if certain additional criteria are fulfilled, an increment of +0.5 is added. The assessment is focused on the appearance of the matrix and cells, and a higher grade suggests more advanced OA.

**Meniscus scoring**

In the Pauli scoring and grading system (2), six individual features are addressed in the microscopic assessment of menisci: specifically, vertical sections (Figure 1B) are used to assess the level of degeneration of *i)* the femoral surface, *ii)* the tibial surface and *iii)* the inner border, while horizontal sections are used to assess *iv)* cellularity and *v)* collagen organization. Further*, vi)* matrix staining intensity of Safranin-O is used to assess proteoglycan content. Each individual feature receives a score from 0 to 3 (on a discrete scale), and in the end, the scores from all six features are summed up to get a cumulative score for each meniscus, which can range from 0 to 18.

**Protocol for histology**

Protocol is based on (3), with some adjustments.

Solutions:

0.1 g Fast Green per 100 mL distilled water

0.1 g Safranin-O per 100 mL distilled water

1 mL acetic acid, glacial in 99 mL distilled water

Weigert’s iron hematoxylin: mix equal part of stock solution A and B.

- Stock solution A: 1 g hematoxylin per 100 mL 95% ethanol
- Stock solution B: 1 mL 29% Ferric chloride in distilled water mixed with 2 mL 37% HCl in 190 mL distilled water. We filtered stock solution B after stirring overnight.

Protocol:

1. Deparaffinize and hydrate slides to distilled water:
2. Xylene 4 min
3. Xylene 4 min
4. Absolute alcohol 4 min
5. Absolute alcohol 4 min
6. 96% alcohol 4 min
7. 70% alcohol 2 min
8. Distilled water 2 min
9. Stain with Weigert’s iron hematoxylin working solution for 10 min
10. Wash in running tap water for 10 min
11. Stain with Fast-Green solution for 5 min
12. Rinse quickly with 1% acetic acid solution for 10-15 s
13. Stain with Safranin-O solution for 5 min
14. Dehydrate and clear
    1. Absolute alcohol 3 min
    2. Absolute alcohol 3 min
    3. Absolute alcohol 3 min
    4. Xylene 2 min
    5. Xylene 2 min
15. Mount using resinous medium

**Reference**

1. Pritzker KP, Gay S, Jimenez SA, Ostergaard K, Pelletier JP, Revell PA, et al. Osteoarthritis cartilage histopathology: grading and staging. Osteoarthritis Cartilage. 2006;14(1):13-29.

2. Pauli C, Grogan SP, Patil S, Otsuki S, Hasegawa A, Koziol J, et al. Macroscopic and histopathologic analysis of human knee menisci in aging and osteoarthritis. Osteoarthritis Cartilage. 2011;19(9):1132-41.

3. Schmitz N, Laverty S, Kraus VB, Aigner T. Basic methods in histopathology of joint tissues. Osteoarthritis Cartilage. 2010;18 Suppl 3:S113-6.
